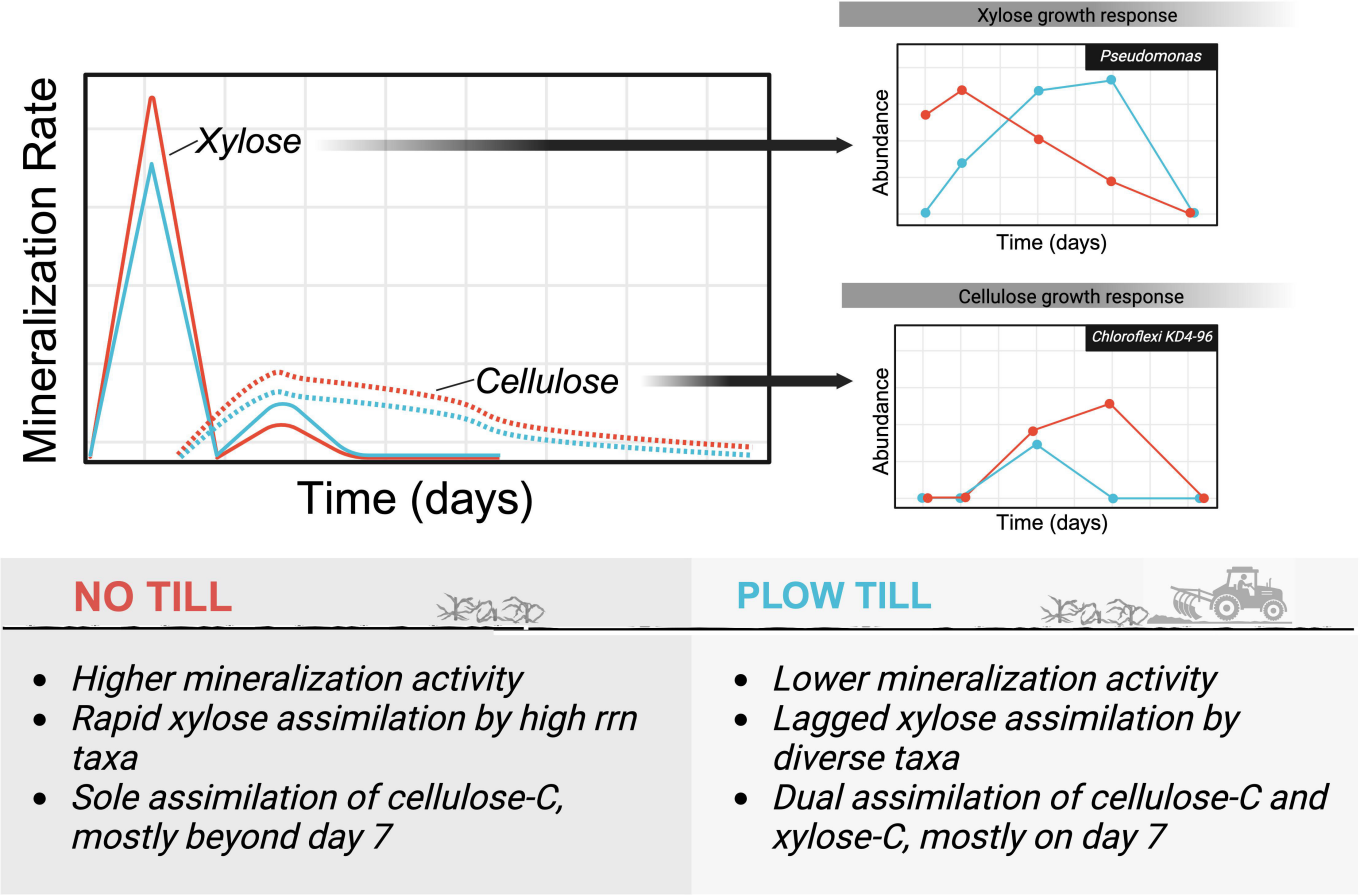# Articles of Significant Interest in This Issue

**DOI:** 10.1128/aem.01785-25

**Published:** 2025-09-17

## A HITCHHIKER’S GUIDE TO POLYHYDROXYALKANOATES 

Polyhydroxyalkanoates are a diverse class of microbially synthesized polymers for applications such as bioplastics. Conners and Bose (e00274-25) review available methods for their quantification and make recommendations for best practices and future directions. 


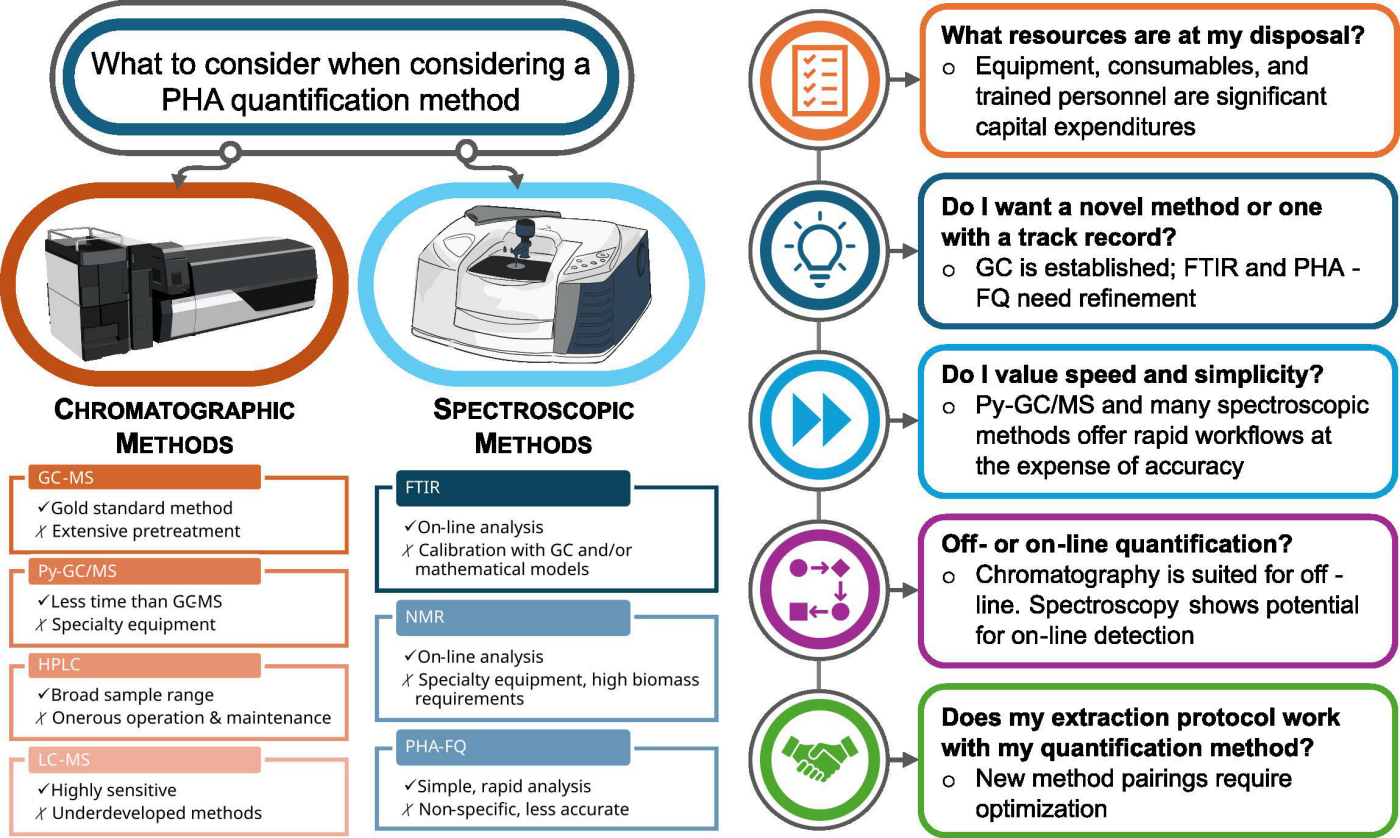


## THE STINKY AROMA OF MYXOBACTERIAL PREDATORS IN AQUACULTURE 

Södergren et al. (e00757-25) reveal the isolation of “stinky” myxobacterial predators from recirculating water of aquaculture systems and the characterization of geosmin and other off-flavored compounds they produce. Knowledge of conditions that promote off-flavored production can guide mitigation controls. 


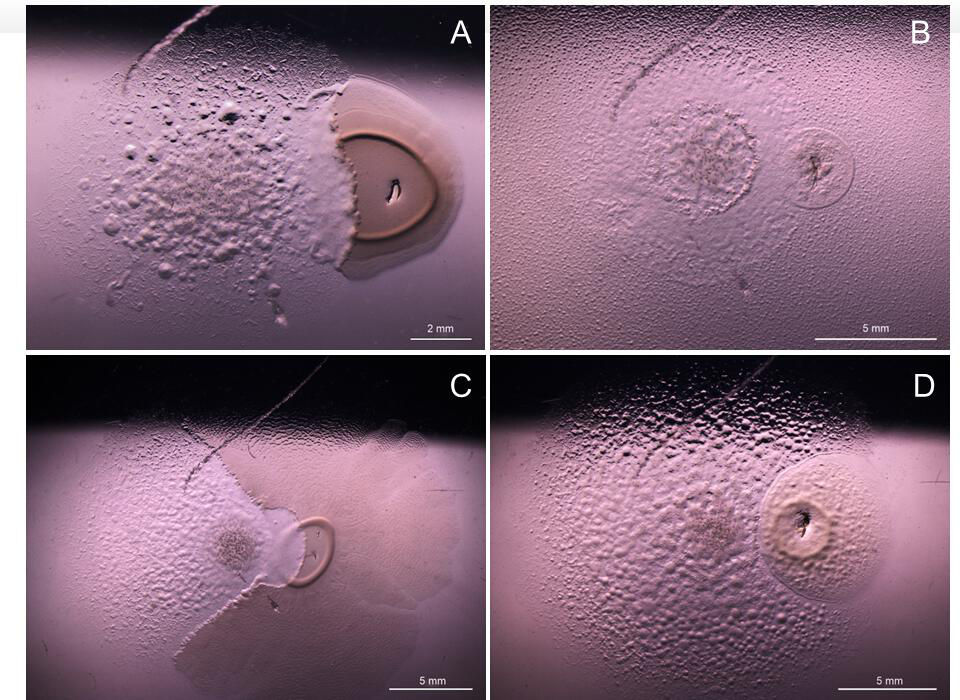


## A LITTLE ACID TO PROTECT THE GUT FROM INFECTIONS 

The acidification of specific gut regions in the fruit fly provides protection from infections, as revealed in this article by Yang et al. (e00707-25). Such a protective priority effect is the result of synergistic interactions between resident microbes and their host.


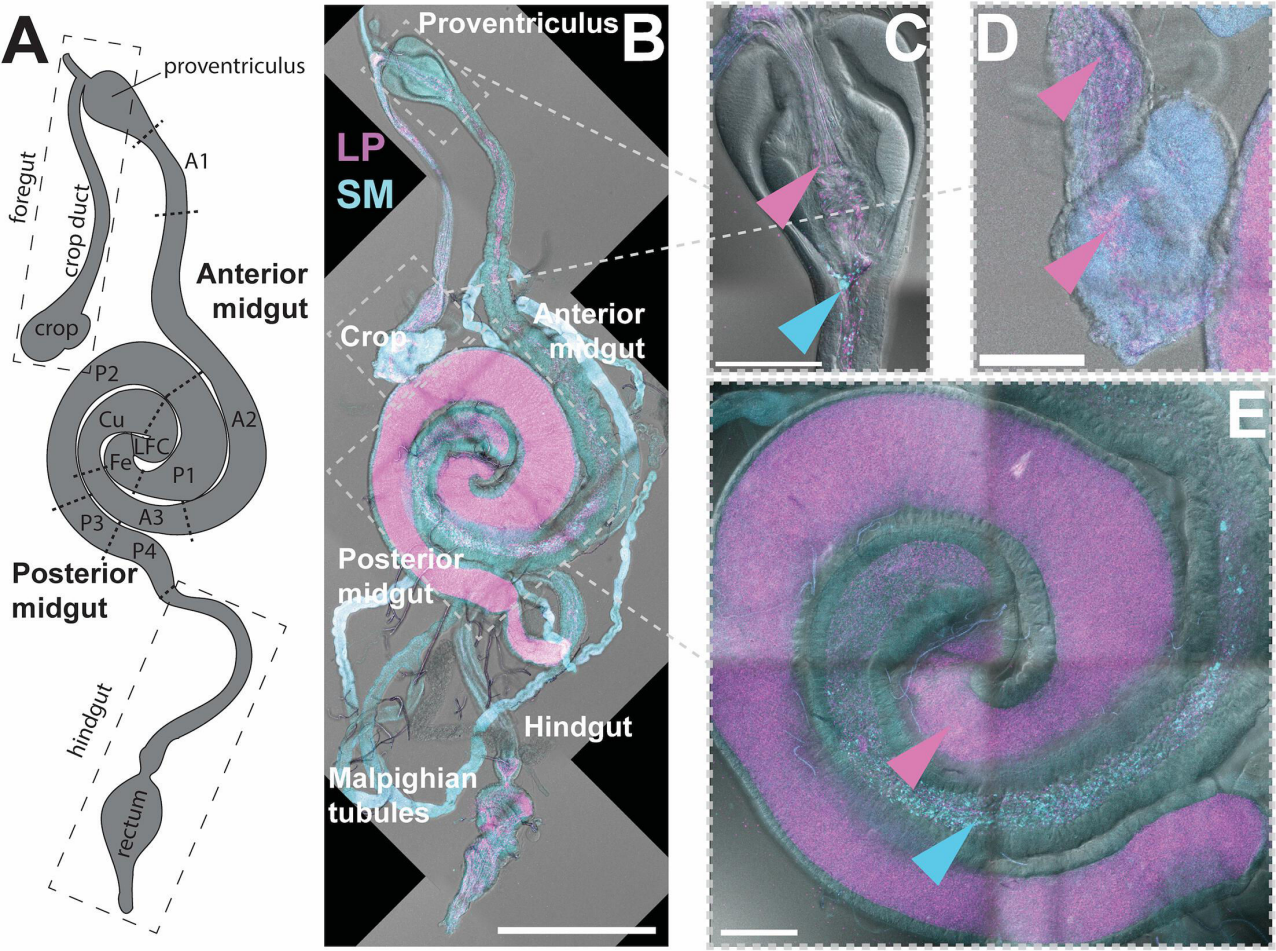


## KILL SWITCHES FOR BIOCONTAINMENT OF INDUSTRIAL STRAINS 

Lamb et al. (e00741-25) developed a biocontainment platform for yeasts via transactivated kill switches. The design rules are widely applicable to other microbes for broad industrial adoption as biocontainment tools. 


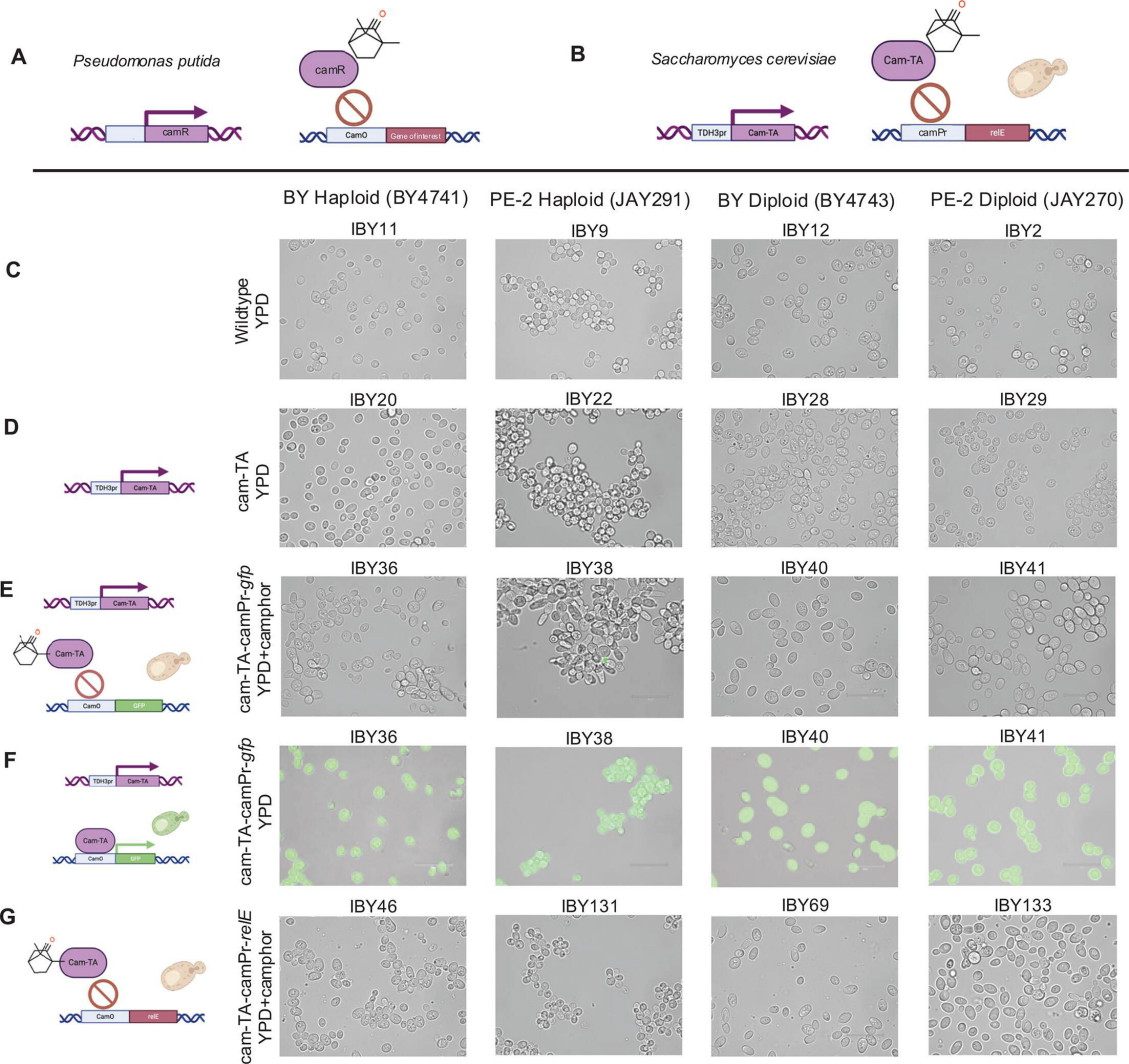


## PHAGE ENGINEERING, CRISPR STYLE 

This article by Fernbach and colleagues (e02014-24) describes a CRISPR approach to engineer staphylococcal phages for precision diagnostics of *Staphylococcus aureus*. This approach has the potential to extend beyond diagnostics, enabling applications of the engineered phages in precision antimicrobial therapies. 


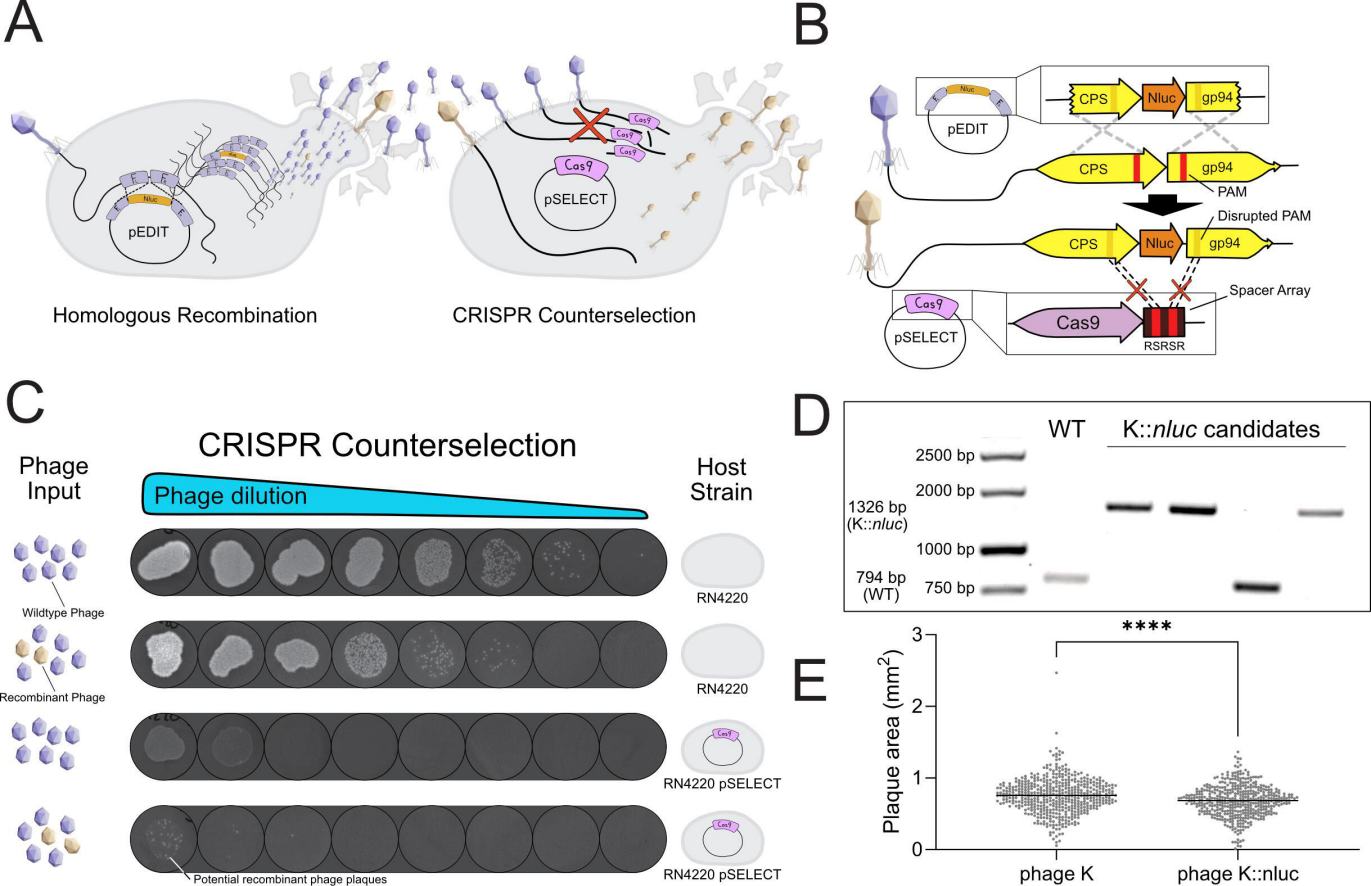


## THE UNEXPECTED IMPACTS OF TILLAGE LEGACY ON SOIL MICROBIOMES 

Agricultural practices such as tillage disturb the soils and their microbial communities, promoting carbon loss. Schaedel et al. (e00933-25) show that tillage legacy alters bacterial community structure and delays the growth of bacteria involved in carbon mineralization.